# Complement-Targeted Therapies in Glioblastoma: A Systematic Review

**DOI:** 10.3390/biomedicines14081702

**Published:** 2026-07-29

**Authors:** Chase M. Walton, Ben A. Strickland

**Affiliations:** Department of Neurosurgery, Medical University of South Carolina, Charleston, SC 29425, USA; waltonch@musc.edu

**Keywords:** glioblastoma, complement system, immunotherapy, systematic review, tumor microenvironment

## Abstract

**Background/Objectives:** Glioblastoma (GBM) is the most common and lethal primary malignant brain tumor in adults, with a median survival of approximately 15 months despite maximal multimodal therapy. The complement system plays a paradoxical dual role in GBM, mediating both antitumor immunity and immunosuppressive signaling within the tumor microenvironment, yet no systematic synthesis of complement-targeted therapeutic strategies exists. We aimed to comprehensively identify, appraise, and synthesize studies investigating complement-targeted therapies and complement-associated prognosis in GBM. **Methods:** Following PRISMA 2020 guidelines, we searched PubMed/MEDLINE and the Cochrane Library (CENTRAL) without date or language restrictions. Preclinical and clinical study designs were eligible. Risk of bias was assessed using SYRCLE, ROBINS-I, and study-type-specific checklists. Certainty of evidence was evaluated using GRADE. Statistical pooling was planned only for sufficiently comparable studies; clinical prognostic studies were synthesized narratively because they assessed non-equivalent constructs. **Results:** Forty-one studies were included, comprising 15 preclinical in vivo, 13 preclinical in vitro, 6 clinical observational, and 7 bioinformatics studies. Five preclinical survival studies entered a structured quantitative synthesis, but no pooled cross-target effect was calculated because their interventions, comparators, and reported summary measures were non-equivalent. Clinical prognostic studies evaluated either individual protein biomarkers or multigene immune-risk signatures and were not pooled. GRADE certainty was “Very Low” for both outcomes. C3b opsonization and the C5a/C5aR1 axis were among the most frequently studied targets. **Conclusions:** Complement modulation remains a promising biological hypothesis in GBM rather than evidence for clinical application, and certainty of evidence is very low. Methodological and mechanistic heterogeneity across complement targets underscore the need for standardized preclinical models and randomized clinical trials.

## 1. Introduction

Glioblastoma (GBM) is the most common and aggressive primary malignant brain tumor in adults, accounting for approximately 50% of all malignant central nervous system (CNS) neoplasms [1,2]. Despite advances in multimodal treatment, including maximal surgical resection, radiotherapy, and temozolomide chemotherapy [3], the prognosis remains dismal, with a median overall survival of approximately 14.6 months and a five-year survival rate of only 3–5% [2,4]. The highly infiltrative nature of GBM, which invades along white matter tracts and perineuronal spaces, renders complete surgical resection problematic [5,6]. Temozolomide resistance develops rapidly, driven in part by the immunosuppressive tumor microenvironment (TME) and intrinsic cellular mechanisms, and recurrence is nearly universal [5,7]. Glioblastoma cell-derived exosomes have been shown to induce microglia to produce complement C5, directly promoting chemotherapy resistance [7], while the complement inhibitor CSMD1 has been associated with differential sensitivity to temozolomide across glioma cell lines [8]. While immunotherapies including immune checkpoint inhibitors and chimeric antigen receptor (CAR) T-cell therapy have transformed outcomes in other solid tumors, GBM remains largely refractory to these approaches, owing in part to its profoundly immunosuppressive TME and the complex interplay between innate immune effectors and tumor-intrinsic resistance pathways [4,9]. These limitations underscore the urgent need for novel therapeutic strategies that can overcome the immune evasion mechanisms characteristic of GBM.

The complement system is a central effector arm of innate immunity comprising more than 50 soluble and membrane-bound proteins organized into three activation pathways: the classical, alternative, and lectin pathways [9,10]. The classical pathway is initiated by antigen–antibody complexes activating the C1 complex; the alternative pathway is triggered by spontaneous hydrolysis of C3 and amplification on permissive surfaces; and the lectin pathway is activated by mannose-binding lectin (MBL) and associated serine proteases (MASPs) recognizing carbohydrate moieties on target cells [11,12]. All three pathways converge on the cleavage of C3 into C3a and C3b, leading to formation of C5 convertase, generation of the potent anaphylatoxin C5a, and assembly of the membrane attack complex (MAC, C5b-9) that lyses target cells [9,13]. In cancer, the complement system plays a paradoxical dual role: on one hand, complement activation can mediate tumor cell killing through MAC formation and opsonization [14,15]; on the other hand, complement components, particularly the anaphylatoxins C3a and C5a, can promote tumor growth by fostering an immunosuppressive microenvironment, enhancing angiogenesis, and recruiting myeloid-derived suppressor cells (MDSCs) [16,17,18].

In glioblastoma specifically, complement dysregulation is increasingly recognized as a hallmark of the TME. Complement activation products including C3, C3b, and the terminal complement complex C5b-9 have been detected in tumor tissue, indicating robust local complement activation, while systemic dysregulation is evident through altered circulating complement levels [13]. The anaphylatoxin receptors C3aR and C5aR1 are upregulated in GBM, with C5aR1 expression increasing with tumor grade and correlating with poor patient survival [16,19,20,21]. Simultaneously, GBM cells upregulate complement regulatory proteins (CD46, CD55, and CD59), which protect tumor cells from complement-mediated cytolysis, while soluble inhibitors including factor H and C1-INH further shield GBM cells from immune clearance [1,5,22,23]. This dual pattern of complement activation and complement evasion within the GBM microenvironment creates a complex therapeutic landscape that has attracted growing research interest.

Despite this growing body of evidence, no systematic review has synthesized the literature on complement-targeted therapeutic strategies in GBM. Individual studies span diverse models (cell lines, xenografts, syngeneic models, patient cohorts, bioinformatics databases), complement targets (C3, C5a, C5aR1, C1-INH, CD46/CD55/CD59), and endpoints (survival, tumor volume, immune markers), making it difficult to draw overarching conclusions from isolated reports. The objectives of this systematic review are to: (1) comprehensively identify and characterize studies investigating complement modulation in GBM; (2) assess the methodological quality of included studies; (3) synthesize survival and prognostic outcomes where data permit; and (4) identify the most promising complement targets and knowledge gaps to guide future research.

## 2. Methods

This systematic review was conducted in accordance with the Preferred Reporting Items for Systematic Reviews and Meta-Analyses (PRISMA) 2020 guidelines [24]. The review was registered in the Open Science Framework (OSF) Registries (https://doi.org/10.17605/OSF.IO/W2FH3), where the a priori protocol, the per-study extraction dataset, the risk-of-bias assessments, and the analysis code are publicly archived; as detailed in Section 2.8, registration was completed retrospectively, after the searches and analyses had been performed.

### 2.1. Search Strategy

A systematic search was conducted across PubMed/MEDLINE (searched 1 March 2026) and the Cochrane Library (CENTRAL; searched 18 June 2026). The search strategy was developed for PubMed and adapted for each database, combining two concept blocks with the Boolean operator AND:

Block A (Complement): complement system proteins, complement activation, complement pathway, complement cascade, C1q, C1r, C1s, C2, C3, C3a, C3b, C4, C5, C5a, C5b, C5aR1, C3aR, membrane attack complex, factor H, factor B, factor D, properdin, CD46, CD55, CD59, mannose-binding lectin, MASP, ficolin, compstatin, eculizumab, avacopan, C1-inhibitor, complement regulatory proteins (searched as MeSH terms and title/abstract keywords).

Block B (Glioblastoma): glioblastoma, glioblastoma multiforme, GBM, glioma, high-grade glioma, astrocytoma grade IV, brain neoplasms, malignant glioma (searched as MeSH terms and title/abstract keywords).

No date, language, or species restrictions were applied. The combined search yielded 810 records (795 from PubMed/MEDLINE and 15 from Cochrane CENTRAL); after removing 1 duplicate, 809 unique records remained. The complete database search strategies are provided in Appendix A.

### 2.2. Eligibility Criteria

Inclusion criteria: Original research (preclinical or clinical); investigation of complement pathway modulation (pharmacological, genetic, or antibody-based); GBM or high-grade glioma model (human patients, animal models, or cell lines); quantitative outcomes reported (survival, tumor volume, immune markers, gene expression).

Exclusion criteria: Narrative reviews, editorials, letters, or case reports with *n* < 3; non-CNS tumors without a GBM-specific subgroup analysis; no complement-specific intervention or measurement; duplicate publications (earliest version retained). Historical glioblastoma or grade IV astrocytoma diagnoses were accepted as reported by the original authors; retrospective molecular reclassification was not attempted when the molecular information required by contemporary WHO criteria was unavailable [25,26].

### 2.3. Process of Study Selection

**Figure 1:**
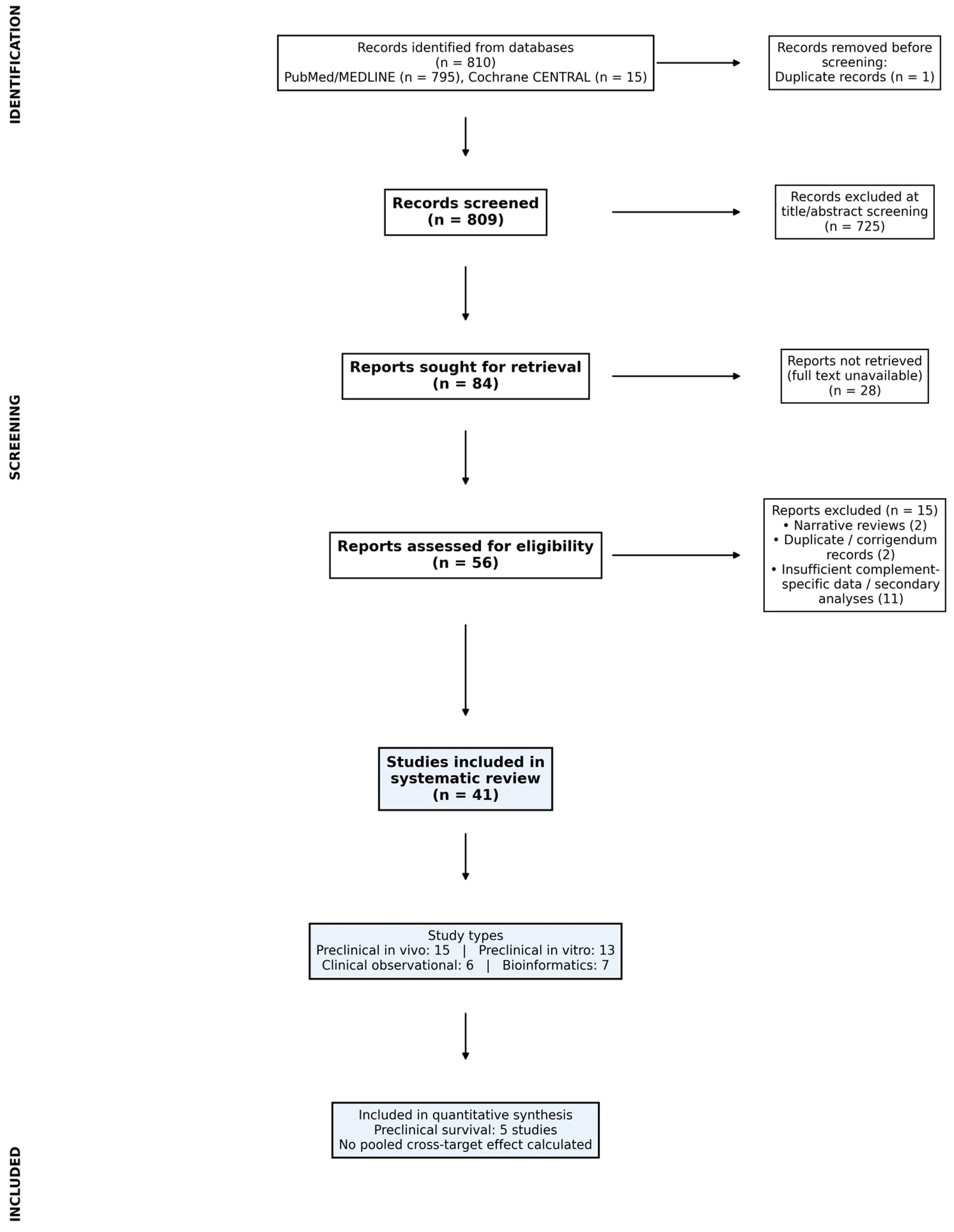
PRISMA 2020 flow diagram illustrating the study selection process. From 809 unique records (810 identified across PubMed/MEDLINE and Cochrane CENTRAL, 1 duplicate removed), 725 were excluded at title/abstract screening, full texts were sought for 84 reports, 56 were retrieved, 28 were not retrieved, 15 of the 56 retrieved reports were excluded, and 41 studies entered the systematic review. Five preclinical survival studies entered the quantitative synthesis.

Study selection followed a two-stage process in accordance with PRISMA 2020 guidelines (Figure 1, File S1). Because complement-related search terms retrieve substantial nephrology and renal transplant literature due to shared biological pathways, a large proportion of the initial records addressed complement biology outside the central nervous system.

Stage 1 (Title/Abstract Screening): All 809 titles and abstracts were screened against the predefined inclusion and exclusion criteria using an AI-assisted workflow comprising automated topical tiering and structured GPT-4o-mini classification (OpenAI; accessed via OpenRouter). Records were excluded if they (a) addressed complement biology in non-CNS contexts (e.g., renal, hepatic, or cardiac pathology) without a glioma-specific component, (b) focused on glioblastoma without any complement-related investigation, (c) were review articles, editorials, or case reports without original data, or (d) did not report relevant outcomes. Structured screening decisions and reasons were logged for reconciliation. A total of 725 records were excluded at this stage, and 84 proceeded to full-text assessment.

Stage 2 (Full-Text Screening): Full texts were sought for the 84 remaining reports; 56 were retrieved and assessed with GPT-4o (OpenAI; accessed via OpenRouter) using a standardized eligibility prompt. Twenty-eight reports were not retrieved despite searches across institutional holdings, open-access repositories, interlibrary loan requests, and direct correspondence with corresponding authors. Fifteen of the 56 retrieved reports were excluded after full-text review for not meeting inclusion criteria (e.g., insufficient complement-specific data, duplicate cohorts, or secondary analyses of previously included datasets). This yielded 41 studies included in the systematic review; five preclinical survival studies entered the quantitative synthesis.

### 2.4. Data Extraction

Candidate data were extracted from the 41 full-text studies with GPT-4o (OpenAI; accessed via OpenRouter) using standardized, study-type-specific forms. The studies included in the systematic review (*n* = 41) are those listed in Table 1 and Table S3; all other cited works are background or methodological references. Records identified as eligible but for which full text could not be retrieved were not carried forward to data extraction or risk-of-bias assessment and are accounted for among the reports not retrieved in the PRISMA flow diagram (Figure 1); two records that were corrigenda or duplicate (preprint) versions of included studies were likewise not double-counted. Three study-type-specific forms were employed:

Preclinical form: study design, species, tumor model, cell lines, complement target, intervention and dose, comparator, tumor volume outcomes, reported survival summary measure (mean or median; treatment vs. control), hazard ratio, immune markers, and key molecular findings.

Clinical/observational form: cohort characteristics, sample size, complement biomarker, measurement method, expression levels, overall survival, hazard ratio with confidence intervals, and multivariate analyses.

Bioinformatics form: databases used, complement genes analyzed, analytical methods, prognostic signatures, survival associations, immune deconvolution results, and external validation.

Model outputs were stored in a structured database and checked for schema completeness. During this revision, the quantitative survival and prognostic fields were re-audited against the source reports with assistance from OpenAI Codex and corrected where necessary.

### 2.5. Risk of Bias Assessment

Risk of bias was assessed using validated tools matched to study design: SYRCLE Risk of Bias tool [27] for preclinical in vivo studies (*n* = 15); ROBINS-I [28] for clinical observational studies (*n* = 6); In Vitro Checklist for preclinical in vitro studies (*n* = 13); and Bioinformatics Checklist for computational studies (*n* = 7). The in vitro and bioinformatics checklists were adapted for this review from established reporting and quality criteria for the respective study types and have not themselves been formally validated; their domain-level criteria are provided in Supplementary Table S5 and the complete study-by-study risk-of-bias judgements in Supplementary Table S2. A summary of the risk-of-bias findings is provided in Appendix B.

Domain-level judgments (Low, Some concerns, High, or Unclear risk) and supporting rationales were generated with GPT-4o (OpenAI; accessed via OpenRouter) using tool-specific prompts; these assessments were not independently duplicated by two human reviewers.

### 2.6. Data Synthesis

Five preclinical survival studies entered a structured quantitative synthesis of study-level findings; no cross-target pooled effect was calculated because the studies differed in complement intervention, comparator, model, combination regimen, and reported survival summary. The four candidate clinical prognostic studies were synthesized narratively rather than pooled because they assessed non-equivalent constructs (individual protein biomarkers versus multigene immune-risk signatures).

Preclinical survival findings were tabulated with their study-defined comparator and reported summary measure. Cross-study heterogeneity statistics, prediction intervals, leave-one-out analyses, and publication-bias tests were not calculated because no common estimand was pooled. Narrative synthesis was used for other outcomes.

### 2.7. Certainty of Evidence

The certainty of evidence for each evidence body was assessed using the Grading of Recommendations, Assessment, Development and Evaluations (GRADE) framework [29]. For each outcome, the body of evidence was rated across five domains: risk of bias, inconsistency, indirectness, imprecision, and publication bias. The starting certainty was “Low” for both outcomes, reflecting the observational nature of the clinical/bioinformatics studies and the inherent limitations of preclinical animal evidence for informing clinical decision-making. We note that some GRADE guidance for animal studies suggests starting at “Very Low”; we adopted a “Low” starting point to allow domain-specific downgrading to be explicitly documented, recognizing that the cumulative downgrades for the preclinical outcome (four levels) and the clinical outcome (three levels) both exceed the distance to the GRADE floor of “Very Low.” The final rating of “Very Low” is therefore robust to the choice of starting level.

For risk of bias, downgrading decisions were informed by the study-level assessments described in Section 2.5: SYRCLE judgments for preclinical studies and ROBINS-I judgments for clinical studies. If the majority of contributing studies had “High” or “Unclear” risk in key domains (randomization, blinding, confounding), the domain was downgraded one level.

For inconsistency, we considered the direction and magnitude of study-level findings and differences in models, comparators, interventions, combination regimens, and reported summary measures. Because no common estimand was pooled, I^2^ and a prediction interval were not used.

For indirectness, we assessed the applicability of the study populations, interventions, and outcomes to the review question. Preclinical evidence was considered very seriously indirect (downgraded two levels) given the uncertain translatability of animal models to human GBM. Clinical evidence was downgraded one level for the heterogeneity of complement biomarkers assessed and the mix of direct clinical observation with retrospective bioinformatics database mining.

For imprecision, we considered the reported uncertainty and sample size within each preclinical study and the completeness of study-specific clinical estimates. Publication bias could not be assessed meaningfully because too few independent, comparable studies were available.

GRADE domains were applied to the final evidence bodies using the rules described above; the assessment was not independently duplicated by two human reviewers. The complete domain-by-domain assessment is presented in Supplementary Table S1.

**Table 1:** Characteristics of included studies (*n* = 41).

Study	Study Type	Complement Target	Model/Population	Intervention or Biomarker	Sample Size (Unit)	Primary Outcome
Mangogna 2019 [30]	Bioinformatics	C1q	NR	C1q	multiple datasets; specific numbers not provided	Higher mRNA levels of C1q chains are associated with poor prognosis in gliomas.
Nguyen 2020 [31]	Bioinformatics	C5aR1	NR	C5aR1	*n* = 238 IDH-wildtype glioma samples	Identification of a ‘normal-like’ IDH-WT glioma subgroup with prolonged survival and distinct immune microenvironment.
Patel 2024 [32]	Bioinformatics	CD59	NR	CD59	Data from TCGA and other public databases	High CD59 expression is associated with poor prognosis in several cancers, including GBM, due to immune evasion.
Qiu 2020 [33]	Bioinformatics	C5aR1	NR	C5aR1	*n* = 1261 glioma cases	The PROMISE model stratifies glioma patients with distinct survival outcomes and highlights immune-related pathways.
Wang 2022 [6]	Bioinformatics	C1R	NR	C1R	169 GBM tumor samples and 1151 non-tumor samples	High expression of C1R, CCL2, and TNFRSF1A is associated with poor survival in GBM patients and correlates with immune cell infiltration.
Wu 2023 [34]	Bioinformatics	C5aR1	NR	C5aR1	*n* = 298 high-grade glioma patients	High C5aR1 expression is associated with poor prognosis in high-grade glioma.
Ye 2022 [2]	Bioinformatics	C5aR1	NR	C5aR1	*n* = 167 GBM samples	Three immune-related genes, PPP4C, C5aR1, and IL-10, were identified as prognostic biomarkers for GBM.
Bouwens 2015 [17]	Clinical observational	C1q	NR	C1q	*n* = 71 GBM patients	Serum levels of complement components and their deposition in tumor tissue
Gahoi 2018 [35]	Clinical observational	factor H	NR	factor H	*n* = 20 glioma patients (CSF samples)	identification of differentially expressed proteins in CSF
Michaud 2013 [36]	Clinical observational	MBL2	NR	MBL2	*n* = 143 glioma cases; *n* = 419 controls	risk of glioma
Mäkelä 2012 [37]	Clinical observational	C4d	NR	C4d	*n* = 102 astrocytoma specimens	patient survival
Xu 2015 [38]	Clinical observational	VSIG4	NR	VSIG4	*n* = 160 high-grade glioma patients	overall survival and progression-free survival
Zhang 2018 [15]	Clinical observational	C3	NR	C3	*n* = 72 glioma patients	reduced complement activation and fewer tumor-infiltrating T cells in IDH-mutant gliomas
Ah-Pine 2023 [16]	Preclinical, in vitro	C3aR	patient-derived (human)	TGF-β1 treatment	37 brain tumors	expression of C3aR and VEGF in TAMs
Barnum 1992 [39]	Preclinical, in vitro	C2	cell_line_only (human)	IFN-y treatment	1–2 × 10^7^ cells per experiment	expression levels of C2, factor B, and factor D mRNA and protein
DeCordova 2019 [5]	Preclinical, in vitro	FHR5	patient-derived (human)	Purified FHR5 from primary GBM cells	Three primary GBM cell lines	Inhibition of complement-mediated lysis
Fujita 1995 [11]	Preclinical, in vitro	MBL	cell_line_only (human)	MBP-MASP complex	six glioma cell lines	C3 and C4 consumption
Hasan 2025 [9]	Preclinical, in vitro	C3	cell_line_only (human)	KYNU silencing, HDAC6 inhibitor tubastatin, BET inhibitor apabetalone	NR	Cell viability reduction
Junnikkala 2000 [22]	Preclinical, in vitro	factor H	cell_line_only (human)	anti-factor H mAbs 131X and OX24	multiple cell lines tested	complement-mediated cell lysis
Kuraya 2003 [12]	Preclinical, in vitro	H-ficolin/Hakata antigen	cell_line_only (human)	—	17 cell lines tested, including 5 glioma lines	Expression of complement components in glioma cell lines
Lim 2020 [40]	Preclinical, in vitro	C5a	cell line xenograft (mouse)	anti-C5a antibody	5 mice/group	invasion and migration of GBM cells
Marx 2020 [41]	Preclinical, in vitro	CD46	cell_line_only (human)	dinutuximab beta	Multiple cell lines with varying GD2 expression levels	ADCC and CDC induction
Nakagawa 2002 [42]	Preclinical, in vitro	C1s	cell_line_only (rat)	overexpression of r-Gsp protein	30 independent clones for overexpression studies	expression and secretion of r-Gsp protein
Sayah 1999 [43]	Preclinical, in vitro	C3a	cell_line_only (human)	Stimulation with C3a and C5a anaphylatoxins	Three human astrocytoma cell lines	IL-6 mRNA expression
Tuysuz 2024 [8]	Preclinical, in vitro	CSMD1	cell line xenograft (mouse)	CSMD1 overexpression	1500 patients across three cohorts, multiple cell lines, and mouse models	CSMD1 expression correlates with decreased tumor aggressiveness and improved survival
Watanabe 1995 [44]	Preclinical, in vitro	C2	cell_line_only (human)	IFN-γ stimulation	Not specified, but multiple experiments with cell cultures	synthesis of C2 and factor B
Förnvik 2017 [1]	Preclinical, in vivo	C1-INH	syngeneic (rat)	anti-C1-INH antibodies	5 control rats, 3 rats with human C1-INH antibodies, 4 rats with rat C1-INH antibodies	survival
Förnvik 2018 [45]	Preclinical, in vivo	C1-INH	cell line xenograft (rat)	anti-C1-INH antibody	42 rats across three experimental series	survival and tumor size reduction
Ikeda 2000 [46]	Preclinical, in vivo	C3	cell line xenograft (rat)	cobra venom factor (CVF) and cyclophosphamide (CPA)	athymic rats with three separate glioma xenografts	infection and propagation of HSV within tumors
Kato 2017 [47]	Preclinical, in vivo	complement-dependent cytotoxicity	cell line xenograft (mouse)	LpMab-21 monoclonal antibody	6 mice per group	tumor volume reduction
Krattli 2026 [19]	Preclinical, in vivo	C5aR1	syngeneic (mouse)	C5aR1 antagonist PMX205	12–24 mice/group reported for Figure S4 panels; exact survival-arm *n* not stated	Cognitive function improvement
Li 2014 [14]	Preclinical, in vivo	C3	syngeneic (mouse)	IDO-blocking drugs (DL-1MT, D-1MT, NLG919) with chemo-radiation	varied across experiments, typically 6–12 mice per group	survival enhancement
Li 2021 [7]	Preclinical, in vivo	C5	mixed (mouse)	C5a receptor (C5aR1) antagonist	10 patient samples, multiple cell lines	tumor sensitivity to TMZ chemotherapy
Lim 2017 [48]	Preclinical, in vivo	C5a	patient-derived (human)	C5a neutralizing antibody	Patient-derived samples and mouse xenograft models	invasiveness of GBM cells
Meng 2024 [20]	Preclinical, in vivo	C5aR1	cell line xenograft (mouse)	C5aR1 inhibitor PMX205	12 mice (6 per group)	tumor size reduction and survival
Oh 2025 [21]	Preclinical, in vivo	C5a	cell line xenograft (mouse)	C5aR1 antagonist W54011	31 mice, 96 patient samples	tumor proliferation, stemness, and invasion
Rosberg 2024 [10]	Preclinical, in vivo	C3	GEM (mouse)	C3aR antagonist SB290157	Multiple cell lines and a genetically engineered mouse model	survival of glioma-bearing mice
Smolag 2025 [49]	Preclinical, in vivo	factor H	mixed (mouse)	FH knockdown	unspecified number of mice for in vivo studies	decrease in the number of ICOS+ Tregs and tendency of prolonged survival
Yu 2023 [50]	Preclinical, in vivo	C5aR1	cell line xenograft (human)	PLK3 inhibitor GW843682X	56 patient samples, multiple cell lines, and mouse xenograft models	radioresistance and tumor growth
Zhai 2021 [4]	Preclinical, in vivo	factor H	both (mouse)	IDO overexpression and IDO enzyme-null cDNA	various, including patient samples and mouse models	IDO nonmetabolically increases factor H levels in human GBM
Zhang 2024 [51]	Preclinical, in vivo	C3aR	mixed (mouse)	C3aR inhibitor JR14a	Nfat1 knockout and wild-type mice, patient-derived glioma stem cells	tumor volume and survival time

PMID, complement-pathway classification, and full-text availability are provided in Supplementary Table S3.

### 2.8. Protocol Deviations

The following deviations from the protocol occurred during the review process. First, Embase, Web of Science, Scopus and ClinicalTrials.gov were listed as information sources in the protocol but were not searched; given that 809 unique records were identified across the two databases searched (PubMed/MEDLINE and the Cochrane Library), and that this review focuses on basic science and translational research rather than registered clinical trials, these omissions are unlikely to have materially affected study identification. We judge the risk that these omissions altered the evidence base to be low for the preclinical questions but non-negligible for the clinical and translational question: because ClinicalTrials.gov and grey-literature sources were not searched, unpublished, ongoing, or terminated trials of complement-targeted agents in GBM may have been missed. Together with the absence of any identified randomized trial, this reinforces our conclusion that the clinical evidence base is immature and that the prognostic associations and preclinical survival findings should be regarded as hypothesis-generating rather than definitive. Second, the protocol specified quality-restricted sensitivity analysis; this was not performed because only two studies (4.9%) were rated as “Low” risk of bias, providing insufficient data for a meaningful quality-restricted subgroup. Third, the protocol specified prospective registration in PROSPERO; this was not completed before the review commenced, and the review was instead registered retrospectively in the OSF Registries (https://doi.org/10.17605/OSF.IO/W2FH3; registered 18 June 2026) using the generalized systematic-review template, with the a priori protocol archived unchanged. Collectively, these deviations are unlikely to have materially affected the conclusions, as the most consequential omissions (Scopus, ClinicalTrials.gov) have substantial indexing overlap with the searched databases and this review’s scope is predominantly preclinical.

## 3. Results

### 3.1. Study Selection

The PRISMA flow diagram is presented in Figure 1. The systematic search across PubMed/MEDLINE and the Cochrane Library (CENTRAL) identified 810 records (809 unique after removing 1 duplicate). At Stage 1 (title/abstract screening), 725 records were excluded, the majority because they addressed complement biology in non-CNS contexts, particularly renal and transplant medicine, which share complement pathway terminology. Full texts were sought for 84 reports; 56 were retrieved. Twenty-eight reports were not retrieved, and 15 of the 56 retrieved reports were excluded after full-text review, yielding 41 studies in the systematic review. Five preclinical survival studies entered the quantitative synthesis. The reasons for full-text exclusion are detailed in the PRISMA flow diagram (Figure 1); the two narrative reviews identified during screening were used only as background references and were not counted among the included studies.

### 3.2. Study Characteristics

The 41 included studies spanned publication years from 1992 to 2026, with a marked increase in publications from 2014 onward and a peak in 2024 (Table 1). By study type, the included studies comprised 15 preclinical in vivo (36.6%), 13 preclinical in vitro (31.7%), 6 clinical observational (14.6%), and 7 bioinformatics (17.1%). The most frequently investigated complement targets were C5a and C5aR1 (combined *n* = 10), C3 (*n* = 5), C3aR (*n* = 2), and C1-INH (*n* = 2). Additional targets included CD59, factor H, FHR5, CSMD1, CD46, C1q, and MASP-1/3. Tumor models included GL261 syngeneic mouse models, patient-derived xenografts, established human GBM cell lines (U87, U251, T98G, H2), and clinical cohorts with TCGA/CGGA bioinformatics validation.

### 3.3. Risk of Bias

Quality assessment results for the 41 fully extracted studies are summarized in Supplementary Table S2 and Supplementary Figures S1–S4. Overall risk of bias was judged as “Some concerns” in 22 studies (53.7%), “High” in 17 (41.5%), “Low” in 2 (4.9%).

Among the 15 preclinical in vivo studies assessed with SYRCLE, the most common concern was inadequate reporting of randomization procedures: allocation concealment, random housing, and blinding of caregivers/investigators were “Unclear” in all 15 studies. Incomplete outcome data was at “Low” risk in all 15 studies. Among the 13 in vitro studies, appropriate controls were included in 13 (100%), but blinding of outcome assessment was “Unclear” in 11 (84.6%) and data availability raised “Some concerns” in 10 (76.9%). Among the 6 clinical observational studies assessed with ROBINS-I, bias due to confounding was rated “High” in 4 (66.7%) and bias in selection of the reported result raised “Some concerns” in 6 (100%). Among the 7 bioinformatics studies, dataset appropriateness and statistical methods were rated “Low” risk in all 7, but code/data availability was rated “High” in 2 (28.6%) and “Some concerns” in 4 (57.1%).

### 3.4. Synthesized Outcomes

#### 3.4.1. Preclinical Survival

Five preclinical in vivo studies reported survival outcomes, but their interventions, comparators, and summary measures were not sufficiently comparable for a pooled cross-target effect. Förnvik et al. [1,45] reported mean survival with dispersion in two C1-INH antibody experiments; Zhang et al. [51] reported separate NFAT1-knockout and JR14a pharmacologic comparisons; Krattli et al. [19] reported no survival benefit with PMX205 alone, whereas longer survival occurred in radiotherapy-containing arms; and Li et al. [14] evaluated IDO blockade added to chemoradiation. These five studies were summarized at the study level without a combined effect estimate (Supplementary Table S4).

#### 3.4.2. Clinical Prognostic Associations

Four clinical observational or bioinformatics studies reported candidate prognostic associations. These studies were not pooled because they did not measure a common construct. Wu et al. [34] reported an adjusted HR of 1.503 (95% CI 1.022–2.209) for radiomics-predicted C5aR1 in high-grade glioma, and Xu et al. [38] reported an adjusted HR of 2.199 (95% CI 1.372–3.523) for VSIG4 measured by immunohistochemistry. Qiu et al. [33] evaluated the multigene PROMISE score, which included C5AR1 (GBM-specific HR 1.8, 95% CI 0.97–3.4), whereas Ye et al. [2] reported a continuous gene-level C5AR1 HR of 1.215 without a confidence interval and separately developed a three-gene immune signature. Differences in construct, measurement, adjustment, and partly overlapping TCGA/CGGA datasets precluded a single effect estimate for “complement expression.”

The complement components assessed as prognostic biomarkers included C5aR1, whose elevated expression in GBM tissues compared to normal brain has been consistently reported across TCGA and CGGA databases and is associated with significantly worse survival [20,21]. C5aR1 inhibits ferroptosis in GBM cells through METTL3-dependent m6A methylation of GPX4, providing a mechanistic basis for its prognostic significance [20]. CD59 expression has been linked to immune evasion and poor outcomes in bioinformatics analyses [32], while C1q upregulation correlates with adverse prognosis [30].

#### 3.4.3. Certainty of Evidence (GRADE)

The GRADE assessment rated the certainty of evidence as “Very Low” for both evidence bodies (Supplementary Table S1).

For the preclinical survival evidence, evidence was downgraded one level for risk of bias, one level for inconsistency because interventions, comparators, experimental models, and summary measures were non-equivalent, and two levels for indirectness. Imprecision could not be assessed using a common confidence interval because no pooled effect was calculated, and publication bias could not be formally assessed with only five studies.

For the clinical prognostic evidence, evidence was downgraded one level for risk of bias (ROBINS-I identified high risk of confounding in 66.7% of clinical studies; bioinformatics studies lack prospective design), one level for inconsistency (the studies assessed non-equivalent markers and risk signatures), and one level for indirectness (individual proteins and multigene immune-risk signatures are not the same construct and were assessed across clinical cohorts and retrospective databases). Imprecision and publication bias could not be assessed reliably because only four partly overlapping studies were available and one did not report a confidence interval.

For both outcomes, the cumulative downgrades from the “Low” starting level exceed the number of levels to the GRADE floor of “Very Low” (four downgrades for preclinical, three for clinical). The “Very Low” rating is therefore the same regardless of whether the starting certainty is set at “Low” (our approach, to permit explicit domain-level documentation) or “Very Low” (as some GRADE guidance for animal and observational evidence suggests).

#### 3.4.4. Tumor Volume and Growth

Insufficient studies reported comparable tumor volume endpoints to permit formal meta-analysis. Standardized mean differences could not be computed because the contributing studies reported tumor burden as percentage reductions or as representative images rather than as group means with standard deviations and per-arm sample sizes; these outcomes are therefore summarized narratively and were not pooled. Narrative synthesis of individual studies indicated that complement-targeted interventions reduced tumor burden in several preclinical models. Anti-C1-INH treatment reduced tumor volume and prolonged survival in glioblastoma-bearing rats [45]. C5aR1 inhibition with PMX205 significantly reduced glioma growth in xenograft models [19]. IDO pathway blockade enhanced C3-dependent tumor destruction in the GL261 model [14]. The anti-GD2 antibody dinutuximab beta mediated complement-dependent cytotoxicity against GBM cells in vitro, though efficacy was limited by expression of complement regulatory proteins [41].

#### 3.4.5. Mechanistic and Molecular Findings

Among the most extensively characterized mechanistic pathways, C3b-mediated opsonization and the C5a/C5aR1 axis have attracted considerable attention. C5a activates C5aR1 signaling to promote GBM cell proliferation, stemness, and invasion [21]. Tumor-associated mesenchymal stem-like cells secrete C5a, which activates ERK MAPK signaling and promotes a hyaluronan-rich microenvironment that enhances GBM invasiveness [48]; the same group subsequently identified the C5a/p38/ZEB1 axis as a key driver of this invasive phenotype [40]. C5aR1 expression is upregulated with increasing glioma grade and correlates with poor prognosis across multiple independent datasets [19,20]. Pharmacological inhibition produced target- and model-specific findings: W54011 reduced tumor growth and improved survival, whereas PMX205 reduced tumor growth and did not compromise radiotherapy efficacy but did not improve survival as monotherapy [19,21].

Complement-dependent cytotoxicity (CDC) represents a distinct therapeutic mechanism. GBM cells exhibit marked resistance to CDC due to high expression of complement regulatory proteins CD46, CD55, and CD59, which inhibit MAC formation [22,23]. CD59 is particularly strongly expressed across glioma cell lines [1,22]. Strategies to overcome this resistance include targeting C1-INH with specific antibodies to reactivate complement-mediated killing [45] and combining anti-tumor antibodies such as dinutuximab beta with approaches to neutralize complement regulators [41].

The lectin pathway has received comparatively less attention but shows relevance to GBM biology. Glioma cell lines express MASP-1 and MASP-3, key enzymes of the lectin complement pathway, and their expression is upregulated during astrocytic differentiation [12,52]. Mannose-binding lectin recognizes glioma cells and can activate complement in vitro [11].

Hypoxia within the GBM microenvironment exacerbates complement dysregulation by promoting C3 expression, driving aggressive tumor phenotypes [10]. GBM cell-derived exosomes containing lncRNA induce microglia to produce complement C5, promoting chemotherapy resistance [7]. Complement factor H functions as an ICOS ligand modulating regulatory T cells in the glioma microenvironment, representing yet another axis of complement-mediated immune modulation [49].

### 3.5. Text Mining and Bibliometric Insights

Exploratory text-mining and bibliometric analyses of the broader literature corpus (TF-IDF keyword extraction, topic modeling, temporal trends, and a complement co-occurrence network) are reported separately from the systematic review, did not inform the evidence synthesis, and are provided in the Supplementary Materials (Figures S5–S9). These exploratory analyses were implemented in Python v3.14 using the scikit-learn [53] and umap-learn [54] packages.

## 4. Discussion

### 4.1. Summary of Findings

This systematic review identified 41 studies investigating complement biology and complement-targeted therapies in glioblastoma, spanning over three decades of research (1992–2026). Five preclinical survival studies were summarized at study level without a pooled cross-target effect because their interventions, comparators, co-interventions, and summary measures were non-equivalent. The four clinical prognostic studies assessed non-equivalent individual biomarkers and multigene signatures and were therefore not pooled. C3b opsonization and the C5a/C5aR1 axis were among the most extensively studied research targets, while complement regulatory proteins (CD46, CD55, CD59) were consistently identified as mediators of resistance to complement-dependent cytotoxicity.

### 4.2. Interpretation

Among the complement components implicated in GBM, C3b-mediated opsonization and the C5a/C5aR1 signaling axis represent two of the more compelling therapeutic targets. C5aR1 is upregulated in GBM tissues relative to normal brain, with expression increasing alongside tumor grade and correlating with poor survival across multiple independent datasets [19,20]. Mechanistically, C5aR1 signaling promotes proliferation, stemness, and invasion through pathways including the C5a/p38/ZEB1 axis and ferroptosis inhibition via METTL3-dependent m6A methylation of GPX4 [20,21,40]. Pharmacological inhibition produced target- and model-specific findings: W54011 reduced tumor growth and improved survival, whereas PMX205 reduced tumor growth and did not compromise radiotherapy efficacy but did not improve survival as monotherapy [19,21].

The potential for synergy between complement modulation and existing immunotherapies deserves particular attention. Complement activation within the GBM microenvironment contributes to immunosuppression through multiple mechanisms, including recruitment of MDSCs, modulation of regulatory T cells via factor H–ICOS interactions, and promotion of an anti-inflammatory cytokine milieu [18,45,49]. Inhibition of C1-INH reactivated complement-mediated cytotoxicity and reduced inflammatory markers (IL-1β, GM-CSF) in glioblastoma-bearing rats [45]. IDO pathway blockade unleashed complement activation leading to C3-dependent tumor destruction, suggesting that combining complement-modulating agents with immune checkpoint inhibitors or metabolic pathway inhibitors could yield synergistic anti-tumor effects [14].

The resistance of GBM cells to complement-mediated cytolysis, mediated by CD46, CD55, and CD59, represents both a challenge and an opportunity. CD59 is strongly expressed across glioma cell lines and provides robust protection against MAC formation [22,23]. Strategies to neutralize these regulatory proteins, whether through blocking antibodies, bispecific constructs, or genetic approaches, could sensitize GBM cells to complement-mediated killing and enhance the efficacy of therapeutic antibodies such as dinutuximab beta [18,41].

### 4.3. Comparison with Existing Literature

Bouwens van der Vlis et al. [18] provided the first comprehensive narrative review of complement activation in GBM, documenting the presence of complement activation products in tumor tissue and serum and highlighting the system’s dual role in tumor biology. Our systematic review extends this work through structured study identification, risk-of-bias assessment, and source-reconciled synthesis of preclinical survival findings. Those findings are reported by study without pooling, and the heterogeneous clinical prognostic studies are likewise reported narratively rather than pooled. The identification of C3b opsonization and the C5a/C5aR1 axis as prominent research targets aligns with recent mechanistic studies but had not previously been established through systematic evidence synthesis. Compared to complement-targeted therapies in other malignancies, where complement activation has been harnessed to enhance antibody-dependent tumor killing [17], the GBM literature reveals a more complex picture in which complement both promotes and inhibits tumor progression depending on the specific components and context involved.

### 4.4. Limitations

Several limitations should be considered when interpreting these findings. First, although the search covered PubMed/MEDLINE and the Cochrane Library (CENTRAL), other major databases (Embase, Web of Science, Scopus), grey literature, and conference abstracts were not searched, and relevant unpublished studies may have been missed. Second, 28 of 84 reports sought after title/abstract screening were not retrieved despite interlibrary loan requests and author contact; this 33% attrition rate may have introduced selection bias, and its direction and magnitude are unknown. Older, non-English, negative, or null reports may be underrepresented. Third, the five preclinical survival studies differed in interventions, comparators, animal models, combination regimens, and reported summary measures, so no common effect was pooled. Fourth, the majority of studies were preclinical, and the clinical evidence is limited to observational and bioinformatics studies; no randomized controlled trials of complement-targeted therapies in GBM were identified. Fifth, only four partly overlapping clinical prognostic studies were available, precluding a meaningful pooled effect or reliable assessment of publication bias. Finally, risk of bias was rated as “High” or “Some concerns” in the vast majority of studies (95.1%), primarily due to inadequate reporting of randomization and blinding procedures in preclinical studies and confounding in observational studies. In addition, AI-assisted screening, extraction, and risk-of-bias appraisal were not independently duplicated by two human reviewers; model error may therefore have affected eligibility decisions, classifications, or extracted fields despite structured prompts and the source-level re-audit of quantitative inputs. Reflecting these limitations, GRADE assessment rated the certainty of evidence as “Very Low” for both the preclinical survival and clinical prognostic outcomes (Section 3.4.3; Supplementary Table S1), indicating that the true effects and associations may differ substantially from the reported findings. Complement modulation should therefore be interpreted as a promising biological hypothesis rather than evidence for clinical application.

Brain tumor classification also changed substantially over the period covered by this review. The 2016 WHO classification introduced integrated molecular diagnoses, and WHO CNS5 (2021) reserves glioblastoma for IDH-wildtype tumors; tumors formerly diagnosed as IDH-mutant glioblastoma are now classified as astrocytoma, IDH-mutant, CNS WHO grade 4, a biologically and prognostically distinct entity [25,26]. Because many older studies lack the molecular data required for retrospective reclassification, their cohorts may mix entities that would now be separated, limiting applicability to contemporary, molecularly defined GBM.

### 4.5. Implications for Future Research

Several critical gaps emerge from this review. Most urgently, no randomized clinical trials of complement-targeted therapies in GBM have been conducted, and the translation of preclinical findings to clinical application remains the primary challenge. The development of standardized endpoints for complement-related studies in GBM, including consensus definitions for complement activation status, standardized survival reporting, and harmonized immune marker panels, would facilitate future meta-analyses and cross-study comparisons.

Specific therapeutic strategies warranting further preclinical investigation include: (1) C5aR1 antagonists as potential radiotherapy adjuncts, with future studies designed to test incremental benefit beyond radiotherapy alone rather than infer synergy from the existing PMX205 comparison [19]; (2) anti-C1-INH antibodies to reactivate complement-mediated tumor killing [45]; (3) combination approaches targeting complement regulatory proteins (CD46, CD55, CD59) to sensitize GBM cells to antibody-dependent complement-mediated cytotoxicity [41]; and (4) integration of complement modulation with immune checkpoint inhibitors and IDO pathway inhibitors [4,14].

The emerging role of CSMD1 as a complement inhibitor with tumor suppressor function in gliomas [8], and the identification of complement factor H as an ICOS ligand modulating regulatory T cells [49], suggest additional therapeutic axes that merit investigation. Radiomics-based prediction of C5aR1 expression levels [34] could enable non-invasive patient stratification for complement-targeted therapies. Table 2 summarizes, by complement target, the strongest available evidence, the principal knowledge gaps, and suggested next steps toward clinical translation.

## 5. Conclusions

This systematic review provides a structured synthesis of complement-targeted research in glioblastoma. Neither the preclinical survival findings nor the non-equivalent clinical prognostic studies were sufficiently comparable for pooling; GRADE certainty is “Very Low,” and no randomized clinical trial was identified. Accordingly, complement modulation remains a promising biological hypothesis rather than robust evidence for clinical application. Given the very low certainty of the current evidence, priorities for future work are standardized preclinical models, target-specific validation of the most promising axes (notably C3b opsonization and the C5a/C5aR1 axis), and strategies to overcome complement regulatory protein–mediated resistance; robust evidence from these steps would be required before complement-targeted clinical trials in GBM could be appropriately designed.

## Figures and Tables

**Table 2:** Clinical-translation readiness of complement targets in glioblastoma, summarizing the strongest available evidence, principal gaps, and suggested next steps by target.

Complement Target/Axis	Highest Evidence Tier	Representative Evidence	Principal Gap	Suggested Next Step
C5a/C5aR1	Preclinical in vivo + prognostic (clinical/bioinformatics)	W54011 reduced tumor growth and improved survival; PMX205 reduced growth and preserved radiotherapy efficacy but did not improve survival alone; C5aR1-related measures were associated with worse OS in observational datasets [2,19,20,21,33,34]	No prospective clinical data; expression cut-offs unstandardized	Test incremental benefit beyond radiotherapy alone in standardized preclinical models before clinical translation
C3/C3b (incl. IDO–complement)	Preclinical in vivo	IDO blockade unleashes C3-dependent tumor killing [14]; hypoxia-driven C3 promotes aggressiveness [10]	Mechanistic only; no clinical translation	Combination preclinical studies (complement + IDO/checkpoint inhibitor) with standardized survival endpoints
C3aR	Preclinical in vivo	NFAT1–C3a–C3aR feedback loop in TAMs; antagonists (JR14a, SB290157) improve survival [10,51]	Largely single-group data; antagonist selectivity	Independent replication and medicinal-chemistry optimization of selective antagonists
C1-INH (C1 esterase inhibitor)	Preclinical in vivo	Anti-C1-INH antibody reduces tumor volume and prolongs survival [1,45]	Single research group; non-clinical-grade antibody	Independent replication; development of a humanized, clinical-grade antibody
Complement regulators (CD46/CD55/CD59)	Preclinical in vitro	Mediate resistance to complement-dependent cytotoxicity; limit dinutuximab beta CDC [41]	No in vivo efficacy data for regulator neutralization	In vivo proof-of-concept combining regulator neutralization with a therapeutic antibody
factor H	Preclinical in vivo/in vitro	Factor H acts as an ICOS ligand modulating Tregs [49]; shields cells from lysis [22]	Context-dependent (anti- vs. pro-tumor) role unresolved; no targeting agent	Define context-specific role; develop a blocking strategy
Lectin pathway (MASP, MBL, ficolin)	Preclinical in vitro	MASP-1/3 and MBL recognize glioma cells and activate complement in vitro [11,12,52]	Minimal evidence; no therapeutic data	Exploratory mechanistic studies before any target nomination
CSMD1	Preclinical	Complement-inhibitor with tumor-suppressor function in glioma [8]	Early-stage; complex biology	Functional validation as a prognostic biomarker/target

## Data Availability

All data supporting the findings of this systematic review are available within the article and its Supplementary Materials. The per-study extraction dataset, risk-of-bias assessments, and Python code for descriptive and exploratory analyses are provided as Supplementary Data and have been deposited in the Open Science Framework (OSF) repository (DOI: https://doi.org/10.17605/OSF.IO/W2FH3), which also archives the a priori protocol and the review’s retrospective registration.

## Supplementary Materials

The following supporting information can be downloaded at: https://www.mdpi.com/article/10.3390/biomedicines14081702/s1, Figure S1: Risk of bias summary: SYRCLE assessment for preclinical in vivo studies. Figure S2: Risk of bias summary: ROBINS-I assessment for clinical observational studies. Figure S3: Risk of bias summary: In Vitro Checklist for preclinical in vitro studies. Figure S4: Risk of bias summary: Bioinformatics Checklist for computational studies. Figure S5: TF-IDF keyword analysis across the study corpus. Figure S6: Topic model heatmap (NMF, 6 topics). Figure S7: UMAP semantic clustering colored by study type. Figure S8: Temporal publication trends in complement-GBM research (1977–2026; based on the broader text-mining corpus rather than the 41 included studies). Figure S9: Complement co-occurrence network (53 nodes, 167 edges). Table S1: GRADE domain-by-domain certainty of evidence assessment. Table S2: Domain-level risk-of-bias assessment for the included studies. Table S3: Study identifiers (PMID), complement-pathway classification, and full-text availability for the included studies. Table S4: Study-level survival outcomes, comparator definitions, and reported summary measures for five preclinical in vivo studies. Table S5: Domain-level criteria for the in vitro and bioinformatics quality-assessment checklists. Data S1: Per-study extraction dataset (CSV). Code S1: Python code for descriptive and exploratory analyses. File S1: PRISMA 2020 Checklist. The per-study extraction dataset (Data S1) and the descriptive and exploratory analysis code (Code S1) are archived in the Open Science Framework repository (DOI: https://doi.org/10.17605/OSF.IO/W2FH3).

## Abbreviations

The following abbreviations are used in this manuscript:

CAR	Chimeric Antigen Receptor
CDC	Complement-Dependent Cytotoxicity
CGGA	Chinese Glioma Genome Atlas
CI	Confidence Interval
CNS	Central Nervous System
CSMD1	CUB and Sushi Multiple Domains 1
ERK	Extracellular Signal-Regulated Kinase
FHR5	Factor H Related Protein 5
GBM	Glioblastoma
GPX4	Glutathione Peroxidase 4
GRADE	Grading of Recommendations, Assessment, Development and Evaluations
HR	Hazard Ratio
ICOS	Inducible T-cell Costimulator
IDO	Indoleamine 2,3-Dioxygenase
lncRNA	Long Non-coding RNA
MAC	Membrane Attack Complex
MAPK	Mitogen-Activated Protein Kinase
MASPs	Mannose-Binding Lectin-Associated Serine Proteases
MBL	Mannose-Binding Lectin
MDSCs	Myeloid-Derived Suppressor Cells
MeSH	Medical Subject Headings
METTL3	Methyltransferase-Like 3
NFAT1	Nuclear Factor of Activated T Cells 1
NLP	Natural Language Processing
NMF	Non-negative Matrix Factorization
PLK3	Polo-Like Kinase 3
PRISMA	Preferred Reporting Items for Systematic Reviews and Meta-Analyses
ROBINS-I	Risk of Bias in Non-randomized Studies of Interventions
SYRCLE	Systematic Review Centre for Laboratory Animal Experimentation
TCGA	The Cancer Genome Atlas
TF-IDF	Term Frequency–Inverse Document Frequency
TME	Tumor Microenvironment
UMAP	Uniform Manifold Approximation and Projection
VSIG4	V-Set and Immunoglobulin Domain-Containing Protein 4
ZEB1	Zinc Finger E-Box Binding Homeobox 1

## Appendix A

### Full Database Search Strategies

The complete search strategies used for each database are presented below. PubMed/MEDLINE was searched on 1 March 2026 and CENTRAL on 18 June 2026. Two concept blocks were combined using the Boolean operator AND: Block A (complement system terms) and Block B (glioblastoma terms). No date, language, or species restrictions were applied.

**Table A1:** Database-specific search strategies and results.

Database	Search Strategy	Records Retrieved
PubMed/MEDLINE	(complement system proteins[MeSH] OR complement activation[MeSH] OR C3a OR C3b OR C5a OR C5aR1 OR C3aR OR membrane attack complex OR factor H OR CD46 OR CD55 OR CD59 OR mannose-binding lectin OR MASP OR eculizumab OR avacopan OR compstatin) AND (glioblastoma[MeSH] OR glioblastoma multiforme OR GBM OR high-grade glioma OR malignant glioma)	795
Cochrane Library (CENTRAL)	Strategy adapted to Cochrane syntax (MeSH descriptors exploded; title/abstract/keyword terms)	15 (CENTRAL); 810 identified across both databases, 1 duplicate removed, 809 unique

## Appendix B

Risk of bias was assessed with the AI-assisted, tool-specific workflow described in Section 2.5. For the 15 preclinical in vivo studies, SYRCLE identified unclear risk across all studies for allocation concealment, random housing, and blinding of caregivers/investigators, while incomplete outcome data was at low risk in all 15 studies. Among the 6 clinical observational studies assessed with ROBINS-I, bias due to confounding was rated high in 4 studies (66.7%) and bias in selection of the reported result raised some concerns in 6 studies (100%). Among the 7 bioinformatics studies, dataset appropriateness and statistical methods were rated low risk in all 7, but code/data availability was rated high in 2 (28.6%) and some concerns in 4 (57.1%). The complete domain-level risk-of-bias assessments are provided in Supplementary Table S2, and the GRADE evidence profiles in Supplementary Table S1.
