# Supplementary material for: Complement-Targeted Therapies in Glioblastoma: A Systematic Review
Source: Biomedicines. 2026 Jul 29;14(8):1702. doi: 10.3390/biomedicines14081702 (PMC13510105; doi:10.3390/biomedicines14081702)
Supplement: Supplementary file 1 [file biomedicines-14-01702-s001.zip › biomedicines-4430236-supplementary.pdf]

Supplementary Materials

Complement-Targeted Therapies in Glioblastoma: A Systematic Review

Chase M. Walton and Ben A. Strickland

This file contains Supplementary Tables S1–S5 and Supplementary Figures S1–S9 referenced in the main text. The per-study extraction dataset (Data S1) and the descriptive and exploratory analysis code (Code S1) are archived in the Open Science Framework repository (DOI: https://doi.org/10.17605/OSF.IO/W2FH3). The analyzed set comprises 41 included studies.

Table S1. GRADE summary of findings and certainty-of-evidence assessment for the preclinical survival evidence body and the clinical prognostic evidence body.

GRADE domain	Preclinical survival evidence	Clinical prognostic evidence
No. of studies	5	4
Study design	Preclinical in vivo (animal models)	Clinical observational and bioinformatics
Synthesis / effect (95% CI)	Structured study-level synthesis; no pooled cross-target effect	Not pooled; individual estimates summarized narratively because the constructs were non-equivalent
Risk of bias	Serious (–1): SYRCLE identified unclear randomization, allocation concealment, and blinding across studies	Serious (–1): ROBINS-I identified high risk of confounding in 66.7% of clinical studies; bioinformatics studies are inherently retrospective
Inconsistency	Serious (–1): interventions, comparators, co-interventions, models, and reported summary measures were non-equivalent	Serious (–1): individual protein markers and multigene immune-risk signatures yielded non-comparable estimates
Indirectness	Very serious (–2): animal models (GL261, xenografts, rat glioma) require indirect extrapolation to human GBM	Serious (–1): individual proteins and multigene signatures were assessed across clinical cohorts and retrospective databases
Imprecision	Not assessable using a common confidence interval because no effect was pooled; individual studies had very small groups and incomplete uncertainty reporting	No additional downgrade; not reliably assessable because there was no pooled interval and one study did not report a confidence interval
Publication bias	Not assessable: only five non-comparable studies were available	Not assessable: only four partly overlapping studies
Certainty of evidence	Very Low	Very Low

Both evidence bodies started at "Low" certainty to permit explicit domain-level documentation; the preclinical evidence body was downgraded four levels (risk of bias –1, inconsistency –1, indirectness –2) and the clinical outcome three levels (risk of bias –1, inconsistency –1, indirectness –1), both exceeding the distance to the GRADE floor, yielding a final rating of "Very Low." With only five studies in the preclinical synthesis and four partly overlapping clinical prognostic studies, publication bias could not be assessed reliably. Findings are hypothesis-generating rather than confirmatory.

Table S2. Overall risk-of-bias / quality rating for each included study, by assessment tool (n = 41).

Study	Tool	Overall risk of bias	Key High / Some concerns / Unclear domains
Mangogna 2019	Bioinformatics Checklist	Some concerns	Code / data availability; Selective reporting concerns
Nguyen 2020	Bioinformatics Checklist	Low	None of concern
Patel 2024	Bioinformatics Checklist	Some concerns	Validation in independent cohort; Code / data availability; Selective reporting concerns
Qiu 2020	Bioinformatics Checklist	Some concerns	Code / data availability
Wang 2022	Bioinformatics Checklist	High	Validation in independent cohort; Code / data availability; Selective reporting concerns
Wu 2023	Bioinformatics Checklist	Some concerns	Validation in independent cohort; Code / data availability
Ye 2022	Bioinformatics Checklist	Some concerns	Multiple testing correction; Code / data availability; Selective reporting concerns

Bouwens 2015	ROBINS-I	High	Bias due to confounding; Bias in selection of participants; Bias in measurement of outcomes; Bias in selection of the reported result
Gahoi 2018	ROBINS-I	High	Bias due to confounding; Bias in selection of participants; Bias due to missing data; Bias in selection of the reported result
Michaud 2013	ROBINS-I	Some concerns	Bias due to confounding; Bias due to missing data; Bias in selection of the reported result
Mäkelä 2012	ROBINS-I	High	Bias due to confounding; Bias in selection of participants; Bias due to missing data; Bias in selection of the reported result
Xu 2015	ROBINS-I	Some concerns	Bias due to confounding; Bias in measurement of outcomes; Bias in selection of the reported result
Zhang 2018	ROBINS-I	High	Bias due to confounding; Bias in selection of participants; Bias in measurement of outcomes; Bias in selection of the reported result
Ah-Pine 2023	In Vitro Checklist	Some concerns	Data availability / reproducibility
Barnum 1992	In Vitro Checklist	Some concerns	Sample size / replication adequacy; Data availability / reproducibility; Selective reporting concerns
DeCordova 2019	In Vitro Checklist	Some concerns	Sample size / replication adequacy; Statistical methods appropriate; Selective reporting concerns
Fujita 1995	In Vitro Checklist	Some concerns	Data availability / reproducibility
Hasan 2025	In Vitro Checklist	Some concerns	Sample size / replication adequacy; Data availability / reproducibility; Selective reporting concerns
Junnikkala 2000	In Vitro Checklist	Some concerns	Sample size / replication adequacy; Statistical methods appropriate; Data availability / reproducibility
Kuraya 2003	In Vitro Checklist	Some concerns	Sample size / replication adequacy; Blinding of outcome assessment; Statistical methods appropriate; Data availability / reproducibility
Lim 2020	In Vitro Checklist	Some concerns	Sample size / replication adequacy; Data availability / reproducibility; Selective reporting concerns
Marx 2020	In Vitro Checklist	Some concerns	Data availability / reproducibility
Nakagawa 2002	In Vitro Checklist	Some concerns	Sample size / replication adequacy; Blinding of outcome assessment; Statistical methods appropriate; Selective reporting concerns
Sayah 1999	In Vitro Checklist	Some concerns	Statistical methods appropriate; Data availability / reproducibility
Tuysuz 2024	In Vitro Checklist	Low	Selective reporting concerns
Watanabe 1995	In Vitro Checklist	Some concerns	Data availability / reproducibility
Förnvik 2017	SYRCLE	High	Other sources of bias; Randomization, allocation concealment, and blinding reporting (Unclear)
Förnvik 2018	SYRCLE	High	Selective outcome reporting; Randomization, allocation concealment, and blinding reporting (Unclear)
Ikeda 2000	SYRCLE	Some concerns	Randomization, allocation concealment, and blinding reporting (Unclear)
Kato 2017	SYRCLE	High	Randomization, allocation concealment, and blinding reporting (Unclear)
Krattli 2026	SYRCLE	Some concerns	Allocation concealment, random housing, investigator blinding, and random outcome assessment (Unclear)
Li 2014	SYRCLE	High	Randomization, allocation concealment, and blinding reporting (Unclear)
Li 2021	SYRCLE	High	Randomization, allocation concealment, and blinding reporting (Unclear)
Lim 2017	SYRCLE	High	Randomization, allocation concealment, and blinding reporting (Unclear)
Meng 2024	SYRCLE	High	Randomization, allocation concealment, and blinding reporting (Unclear)
Oh 2025	SYRCLE	High	Randomization, allocation concealment, and blinding reporting (Unclear)
Rosberg 2024	SYRCLE	High	Randomization, allocation concealment, and blinding reporting (Unclear)
Smolag 2025	SYRCLE	High	Randomization, allocation concealment, and blinding reporting (Unclear)
Yu 2023	SYRCLE	High	Randomization, allocation concealment, and blinding reporting (Unclear)

Zhai 2021	SYRCLE	High	Randomization, allocation concealment, and blinding reporting (Unclear)
Zhang 2024	SYRCLE	Some concerns	Other sources of bias; Randomization, allocation concealment, and blinding reporting (Unclear)

Preclinical in vivo studies were assessed with SYRCLE, clinical observational studies with ROBINS-I, and preclinical in vitro and bioinformatics studies with checklists developed for this review (criteria in Table S5). For SYRCLE rows, Unclear denotes domains that were not adequately reported; these unresolved domains contributed to the overall judgment even when no domain was explicitly rated High or Some concerns. Domain-level judgements are visualized in Figures S1–S4.

Table S3. Study identifiers (PMID), complement-pathway classification, and full-text availability for the 41 included studies.

Study	Study type	Complement pathway	PMID	Full text
Mangogna 2019	Bioinformatics	Classical	31649675	Available
Nguyen 2020	Bioinformatics	Terminal	33059718	Available
Patel 2024	Bioinformatics	Terminal	39518137	Available
Qiu 2020	Bioinformatics	Regulation	33425732	Available
Wang 2022	Bioinformatics	Multiple	35726234	Available
Wu 2023	Bioinformatics	Terminal	37760630	Available
Ye 2022	Bioinformatics	Terminal	36230961	Available
Bouwens 2015	Clinical observational	Multiple	25468776	Available
Gahoi 2018	Clinical observational	Multiple	28679024	Available
Michaud 2013	Clinical observational	Lectin	23637788	Available
Mäkelä 2012	Clinical observational	Multiple	23199209	Available
Xu 2015	Clinical observational	Regulation	26279760	Available
Zhang 2018	Clinical observational	Multiple	30006485	Available
Ah-Pine 2023	Preclinical, in vitro	Alternative	37174113	Available
Barnum 1992	Preclinical, in vitro	Multiple	1445220	Available
DeCordova 2019	Preclinical, in vitro	Alternative	31519376	Available
Fujita 1995	Preclinical, in vitro	Lectin	7730143	Available
Hasan 2025	Preclinical, in vitro	Multiple	40843669	Available
Junnikkala 2000	Preclinical, in vitro	Alternative	10820293	Available
Kuraya 2003	Preclinical, in vitro	Lectin	12502731	Available
Lim 2020	Preclinical, in vitro	Terminal	32179921	Available
Marx 2020	Preclinical, in vitro	Regulation	32246395	Available
Nakagawa 2002	Preclinical, in vitro	Classical	12393260	Available
Sayah 1999	Preclinical, in vitro	Terminal	10349852	Available
Tuysuz 2024	Preclinical, in vitro	Regulation	38561856	Available
Watanabe 1995	Preclinical, in vitro	Multiple	7832755	Available
Fo¨rnvik 2017	Preclinical, in vivo	Classical	28880870	Available
Förnvik 2018	Preclinical, in vivo	Classical	30647842	Available
Ikeda 2000	Preclinical, in vivo	Multiple	10775615	Available
Kato 2017	Preclinical, in vivo	Terminal	28234556	Available
Krattli 2026	Preclinical, in vivo	Terminal	41085542	Available
Li 2014	Preclinical, in vivo	Multiple	25054064	Available
Li 2021	Preclinical, in vivo	Terminal	34667108	Available
Lim 2017	Preclinical, in vivo	Terminal	27903965	Available
Meng 2024	Preclinical, in vivo	Regulation	39368999	Available
Oh 2025	Preclinical, in vivo	Terminal	41353504	Available
Rosberg 2024	Preclinical, in vivo	Multiple	39172519	Available
Smolag 2025	Preclinical, in vivo	Alternative	39378431	Available
Yu 2023	Preclinical, in vivo	Regulation	37568042	Available
Zhai 2021	Preclinical, in vivo	Regulation	34479957	Available
Zhang 2024	Preclinical, in vivo	Regulation	38289255	Available

All 41 included studies had retrievable full text. Of the 84 reports sought for retrieval, 56 were retrieved and 28 were not; unretrieved reports were excluded before inclusion and are not represented in this table.

Table S4. Source-verified study-level survival findings and reasons for non-pooling (five preclinical in vivo studies).

Study	Target / model	Comparator	Intervention	Reported survival; measure; dispersion; n	Audit finding / reason not pooled
Krattli 2026	C5aR1; syngeneic mouse (GL261 and CT2A)	Vehicle or PMX205 without radiotherapy	Radiotherapy or radiotherapy plus PMX205	GL261 averages 36–39 versus 54–60 days; CT2A averages 35–40 days; dispersion and survival-group n not reported	PMX205 alone did not improve survival; the grouped GL261 contrast primarily captures radiotherapy
Zhang 2024	C3aR; GL261 syngeneic mouse model; Nfat1 backgrounds	Saline: mean 24.8 ± 0.86 days	JR14a: mean 67.8 ± 1.31 days	Mean ± SD; per-arm n not stated	The previously extracted 75.5-versus-22.2-day values were NFAT1-knockout versus wild-type, not JR14a
Förnvik 2018	C1-INH; syngeneic rat; established tumor	Untreated: mean 32.7 ± 2.4 days; PBS: mean 29.3 ± 0.8 days	Intratumoral anti-C1-INH: mean 51.0 ± 7.7 days	Mean with dispersion type not defined; n = 6 per group	Comparator choice changes the numerical contrast
Förnvik 2017	C1-INH; syngeneic rat; tumor-cell pre-coating before implantation	Control: mean 23.0 ± 3.4 days	Anti-human C1-INH: mean 29.7 ± 4.0 days; anti-rat C1-INH: mean 32.8 ± 3.8 days	Mean ± SD; control n = 5, anti-human n = 3, anti-rat n = 4	Pre-implantation cell manipulation, not treatment of established disease
Li 2014	IDO blockade with C3 downstream; syngeneic mouse (GL261)	Temozolomide plus radiotherapy: median 31 days	DL-1MT plus temozolomide and radiotherapy: median 37.5 days	Median; dispersion not reported; control n = 6, intervention n = 8	IDO-targeted intervention rather than direct complement targeting

Values are reproduced using the summary measure reported by each source. Förnvik (2017) reported mean ± SD; Förnvik (2018) reported means with dispersion but did not define the dispersion type; Zhang (2024) reported mean ± SD for JR14a versus saline; Li (2014) reported medians without dispersion; and Krattli (2026) reported grouped average ranges without arm-specific dispersion or survival-group sample sizes. The studies differed in target or mechanism, comparator, co-intervention, model, and summary measure; therefore, no common survival estimand was pooled.

Table S5. Domain-level criteria for the in vitro and bioinformatics quality-assessment checklists.

Checklist	Domain	Criterion assessed (a "Low risk of bias" rating requires…)
In Vitro Checklist	Sample size / replication adequacy	Experiments performed with adequate biological and/or technical replicates and a justified number of independent experiments.
	Appropriate controls included	Suitable positive, negative, and/or vehicle controls included for each assay.
	Blinding of outcome assessment	Outcome assessment performed blind to experimental condition.
	Statistical methods appropriate	Statistical tests appropriate to the data and clearly described, with assumptions met.
	Data availability / reproducibility	Underlying data and sufficiently detailed methods available to permit reproduction.
	Selective reporting concerns	All assayed / pre-specified outcomes reported, without evidence of selective reporting.
Bioinformatics Checklist	Dataset appropriateness and size	Public or primary dataset(s) appropriate to the question, with adequate sample size.
	Validation in independent cohort	Key findings validated in at least one independent cohort or dataset.
	Multiple testing correction	Correction for multiple comparisons applied where relevant.
	Statistical methods appropriate	Survival and/or expression analyses appropriate to the data and clearly specified.
	Code / data availability	Analysis code and processed data publicly available.
	Selective reporting concerns	Outcomes / genes reported without evidence of selective reporting.

Both checklists were developed for this review to capture core rigor and reporting elements relevant to in vitro experimental and computational/bioinformatics prognostic studies, respectively; they are not previously validated instruments. Each domain was rated Low risk, Some concerns, High risk, or Unclear, and an overall judgement was assigned per study (Table S2).

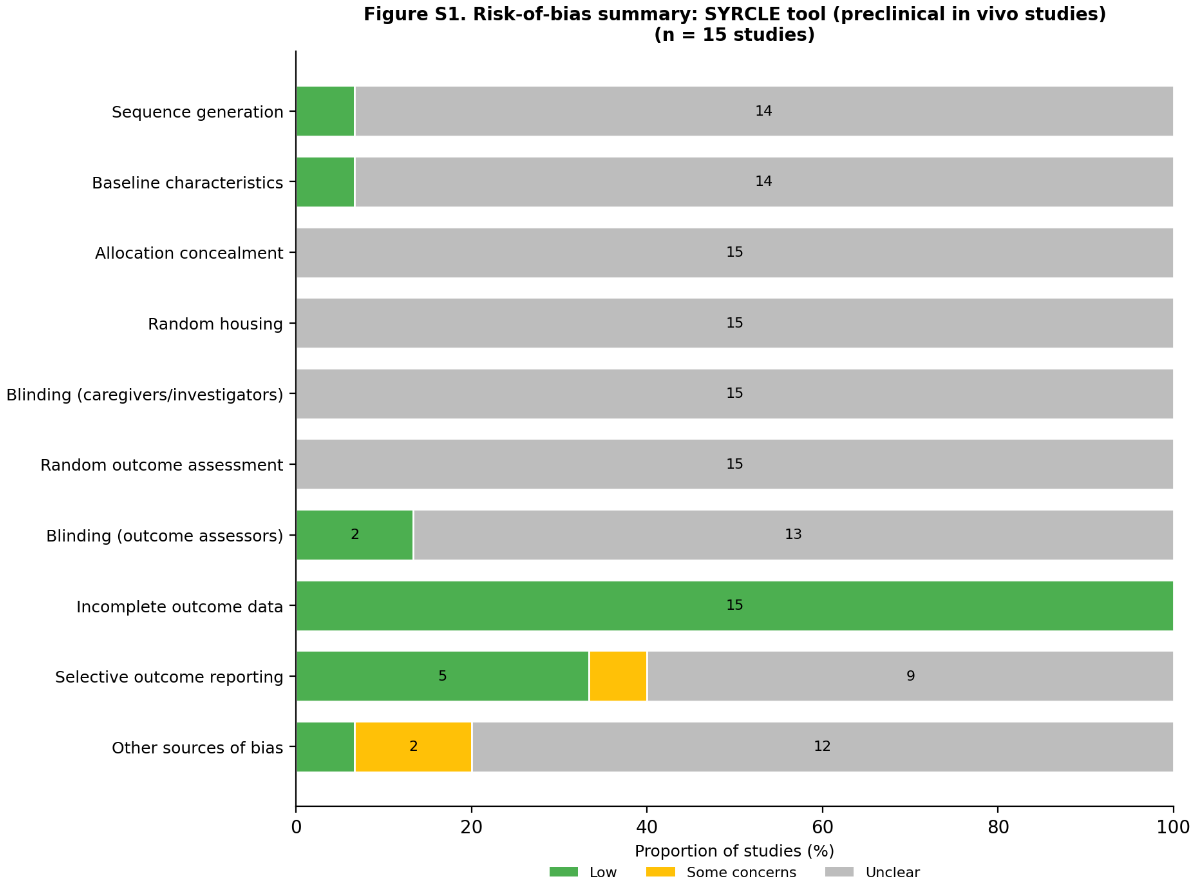

Figure S1. Risk-of-bias summary (SYRCLE) for the preclinical in vivo studies (n = 15). Bars show the proportion of studies rated at each level per domain; counts are annotated.

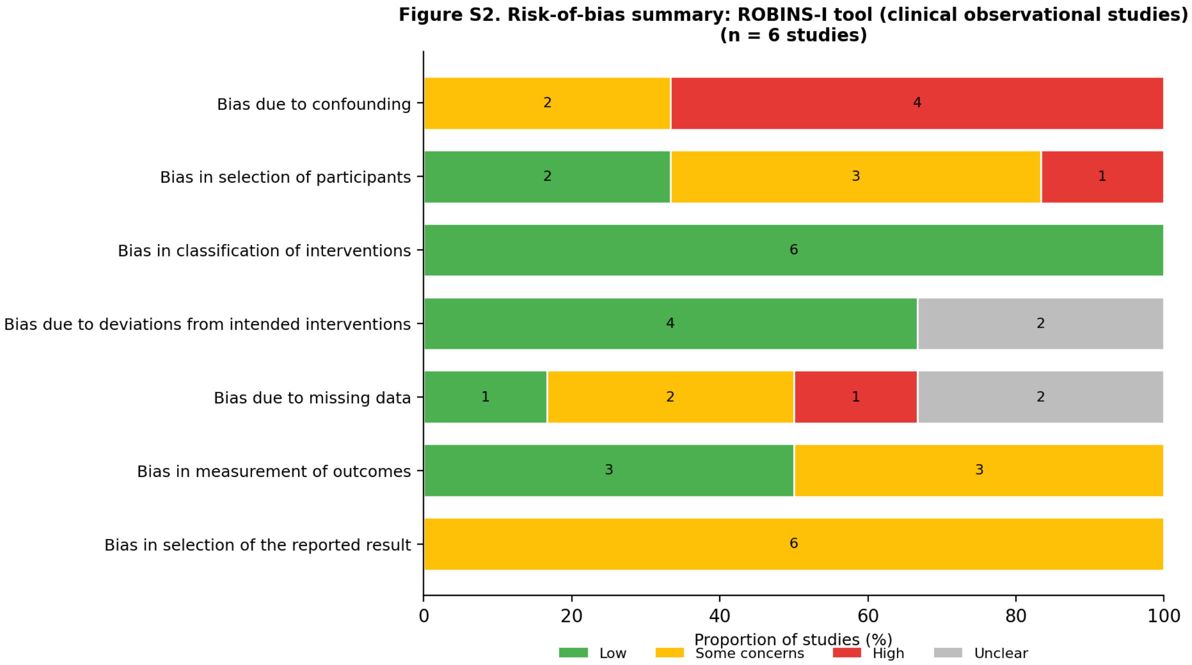

Figure S2. Risk-of-bias summary (ROBINS-I) for the clinical observational studies (n = 6).

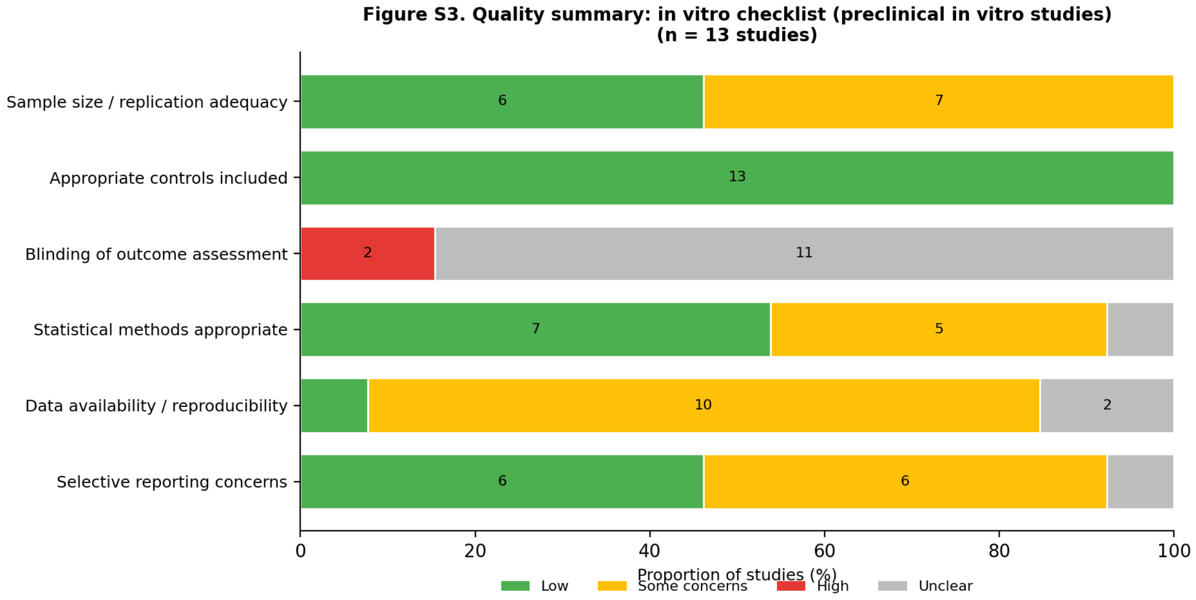

Figure S3. Quality summary (in vitro checklist) for the preclinical in vitro studies (n = 13).

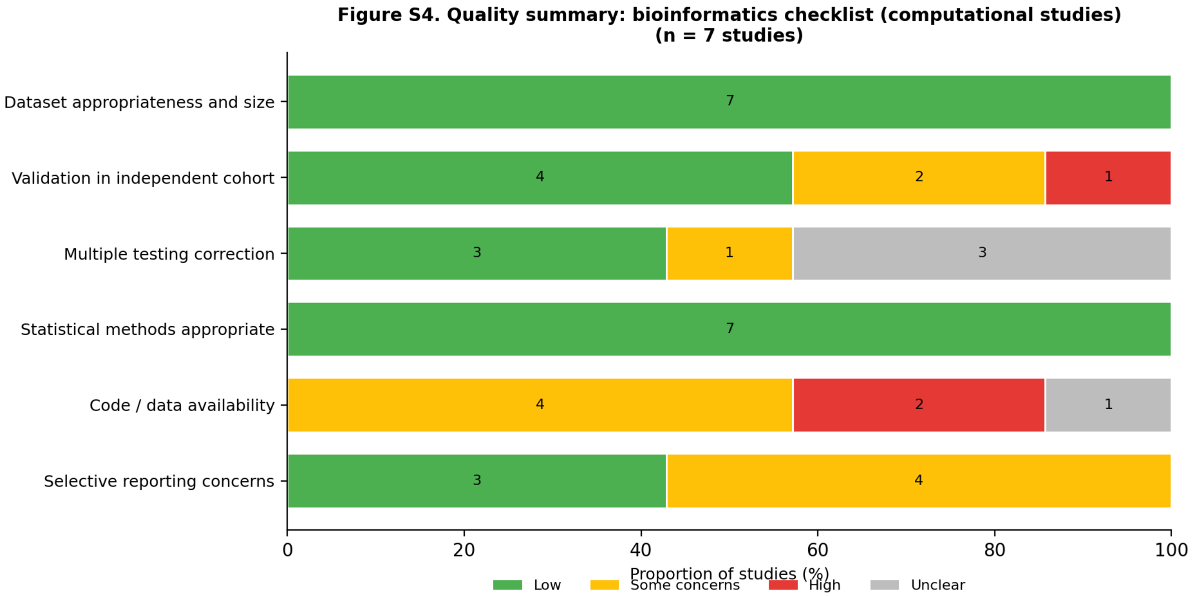

Figure S4. Quality summary (bioinformatics checklist) for the computational studies (n = 7).

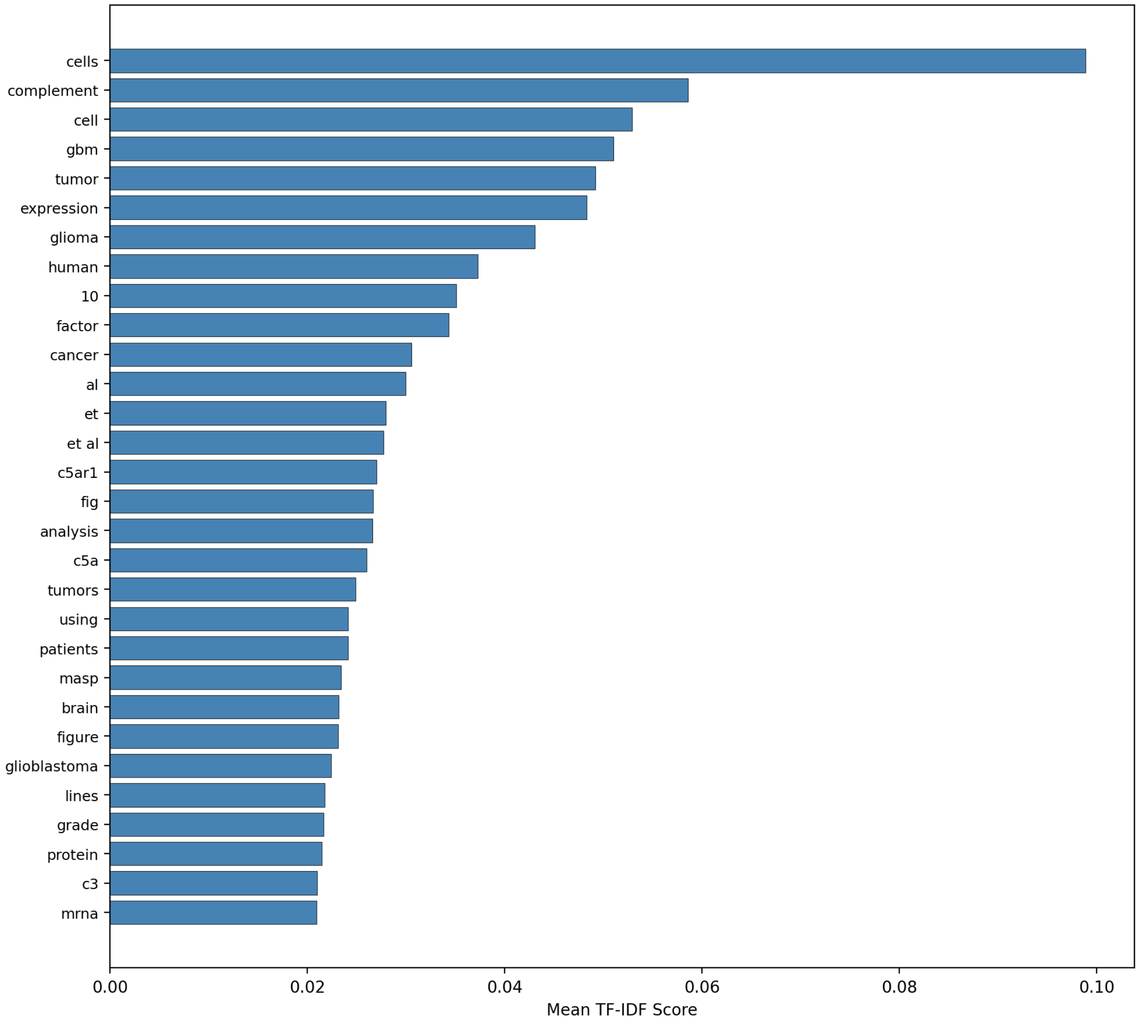

Figure S5. TF-IDF keyword analysis across the study corpus.

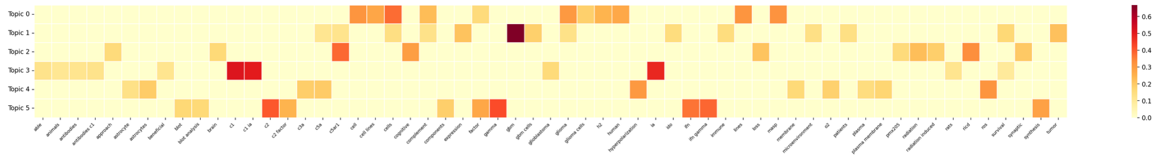

Figure S6. Topic-model heatmap (non-negative matrix factorization, six topics).

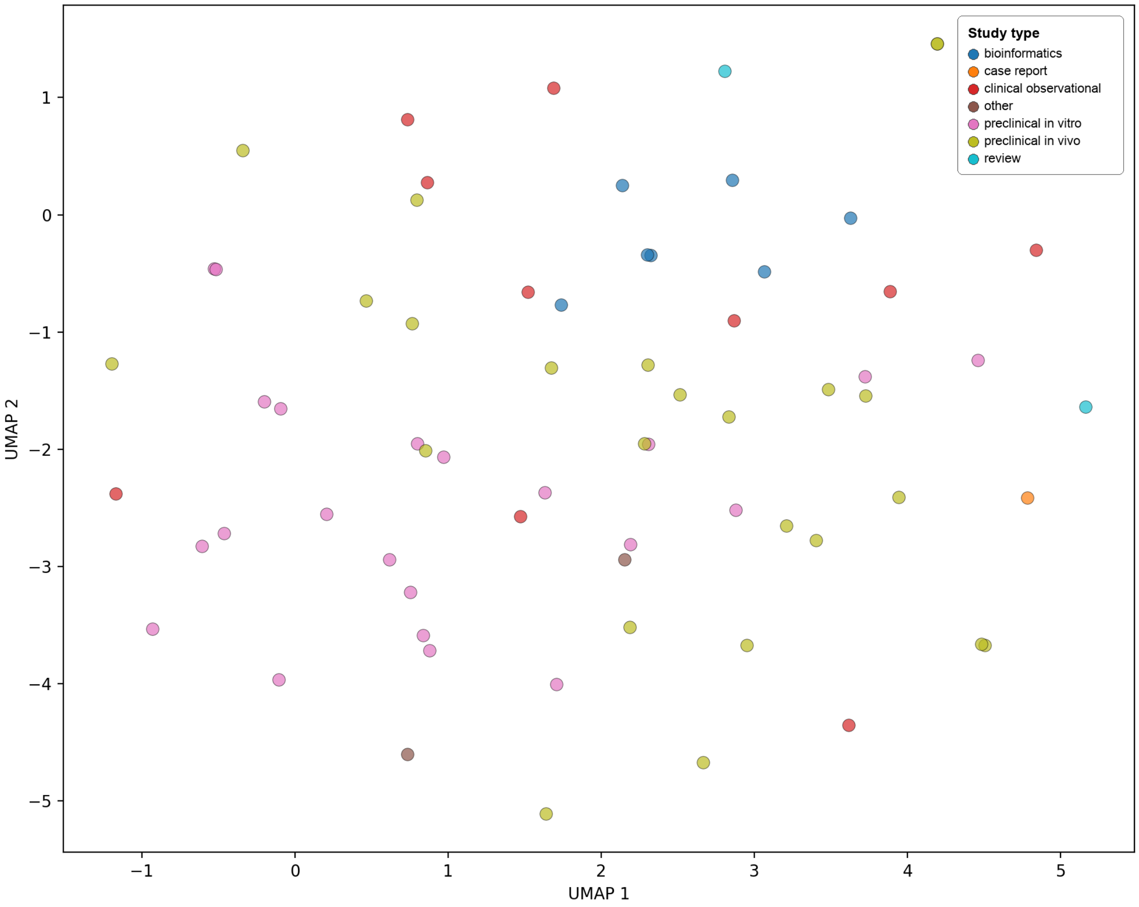

Figure S7. UMAP semantic clustering of the corpus, colored by study type.

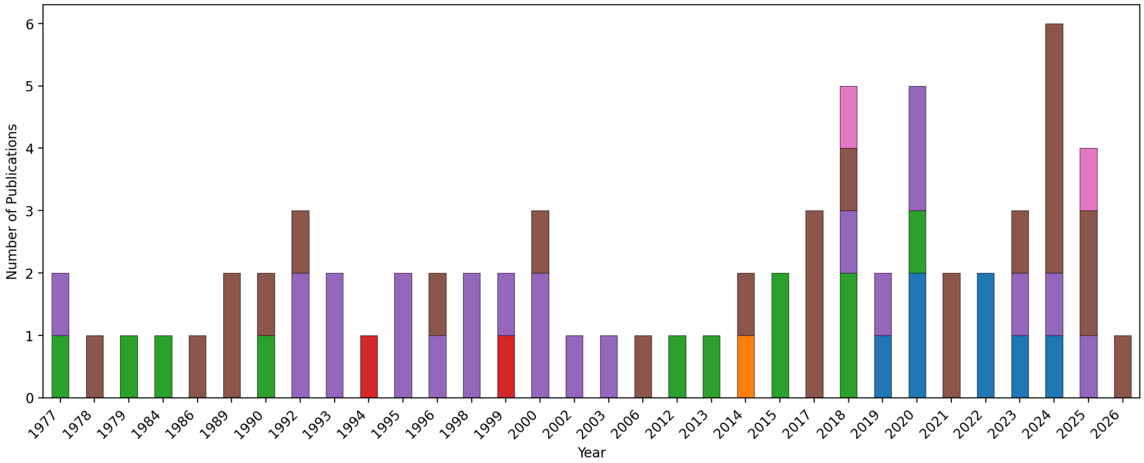

Figure S8. Temporal publication trends in complement–GBM research (based on the broader text-mining corpus rather than the 41 included studies).

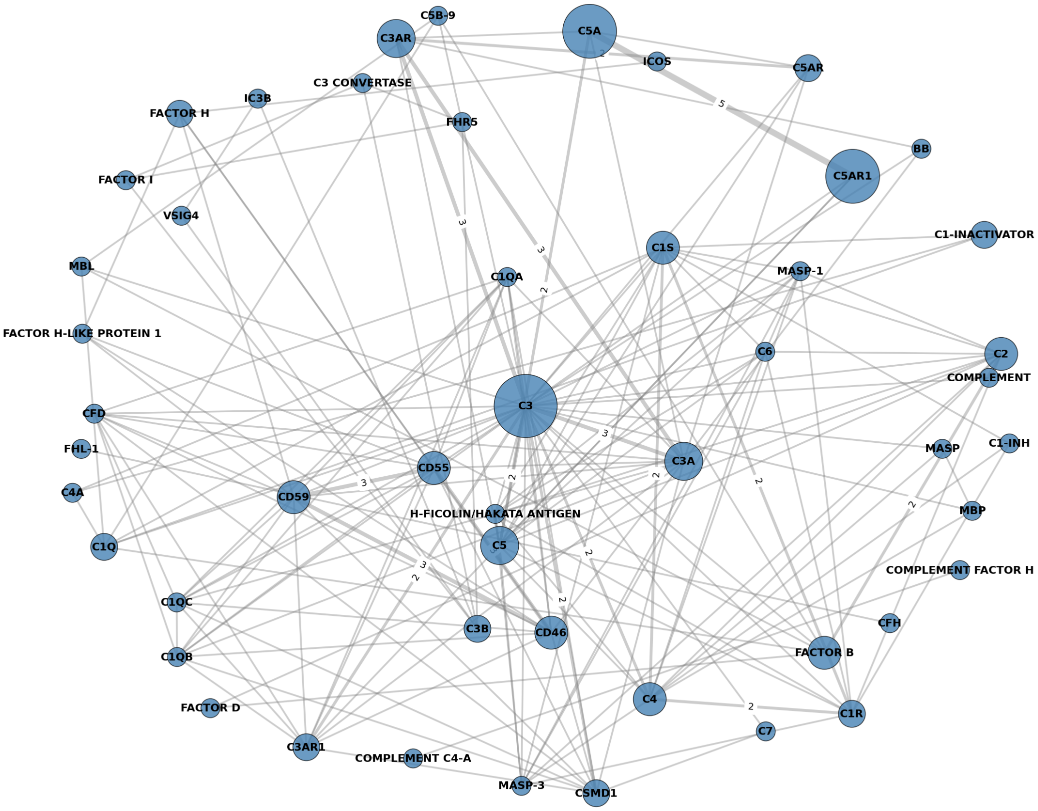

Figure S9. Complement co-occurrence network (53 nodes, 167 edges).

Figures S5–S9 present exploratory text-mining and bibliometric analyses that were reported separately from the systematic review and did not inform the evidence synthesis, and include abstracts from records beyond the 41 included studies. These analyses were implemented in Python using scikit-learn and umap-learn.
